# The Geographic Variation of Surveillance and Zoonotic Spillover Potential of Influenza Viruses in Domestic Poultry and Swine

**DOI:** 10.1093/ofid/ofy318

**Published:** 2018-11-27

**Authors:** Kathryn A Berger, David M Pigott, Francesca Tomlinson, David Godding, Sebastian Maurer-Stroh, Biruhalem Taye, Fernanda L Sirota, Alvin Han, Raphael T C Lee, Vithiagaran Gunalan, Frank Eisenhaber, Simon I Hay, Colin A Russell

**Affiliations:** 1 Department of Veterinary Medicine, University of Cambridge, United Kingdom; 2 Institute for Health Metrics and Evaluation, University of Washington, Seattle; 3 Bioinformatics Institute, A*STAR, Singapore; 4 National University of Singapore; 5 Academic Medical Center, University of Amsterdam, The Netherlands; 6 Agrimetrics Ltd., Harpenden, United Kingdom; 7 European Molecular Biology Laboratory, Deutsches Elektronen-Synchrotron, Hamburg, Germany

**Keywords:** avian influenza, outbreak, spillover, surveillance, swine influenza

## Abstract

**Background:**

Avian and swine influenza viruses circulate worldwide and pose threats to both animal and human health. The design of global surveillance strategies is hindered by information gaps on the geospatial variation in virus emergence potential and existing surveillance efforts.

**Methods:**

We developed a spatial framework to quantify the geographic variation in outbreak emergence potential based on indices of potential for animal-to-human and secondary human-to-human transmission. We then compared our resultant raster model of variation in emergence potential with the global distribution of recent surveillance efforts from 359105 reports of surveillance activities.

**Results:**

Our framework identified regions of Southeast Asia, Eastern Europe, Central America, and sub-Saharan Africa with high potential for influenza virus spillover. In the last 15 years, however, we found that 78.43% and 49.01% of high-risk areas lacked evidence of influenza virus surveillance in swine and domestic poultry, respectively.

**Conclusions:**

Our work highlights priority areas where improved surveillance and outbreak mitigation could enhance pandemic preparedness strategies.

In the last century, only 4 influenza virus subtypes have spread from animals to humans resulting in pandemics; however, these viruses represent a small fraction of the influenza virus subtypes circulating in nature [1, 2]. Animal influenza viruses vary in their likelihood of causing a pandemic, but their vast diversity makes the systematic assessment of the risks posed by each virus subtype logistically infeasible. Current pandemic influenza risk assessments largely focus on viruses with a prior history of animal-to-human transmission under the assumption that they pose a greater threat to human health (eg, A/H5N1 and A/H7N9). However, this intuitive assumption has little empirical support [3].

Surveillance is vital for the early detection of disease threats and the development of more efficient influenza pandemic mitigation strategies [4]. Information on the geographic variation of influenza virus emergence risk and/or spillover, framed within concurrent surveillance efforts, is essential for the design of future influenza surveillance and pandemic mitigation strategies. However, to date, there is no centralized global data repository on animal influenza surveillance. Institutions such as the World Organisation for Animal Health (OIE) have turned to voluntary surveys of veterinary health officers to estimate levels of surveillance in different parts of the world [5]. Existing surveillance efforts vary considerably by host and geographic region, largely in response to prior threats, with only a fraction of the gathered information entering the public domain [3, 5–7]. Furthermore, surveillance in domestic livestock is largely focused on the safeguarding of animal health with regards to its impact on international trade [5], and therefore it does not capture the extent of all animal viruses circulating globally. In poultry, surveillance activities prioritize highly pathogenic avian influenza (HPAI) viruses H5 and H7 subtypes, which require OIE notification, because of their potential for high pathogenicity [8]. Non-H5/H7 influenza A viruses (ie, H1–4, H6, and H8–16) do not require formal reporting [9], allowing a variety of animal viruses to circulate globally unmonitored [10].

The systematic integration of known factors of animal influenza transmission can be used to develop zoonotic risk profiles, providing surveillance targets and situational awareness of conditions that may be indicative of outbreak emergence potential [11, 12]. This approach could alert public health officials of elevated risk conditions and trigger targeted interventions and surveillance activities [11]. The ecological factors associated with the animal-to-human spillover of influenza viruses are subject of debate; most studies aimed at quantifying these factors have focused solely on HPAI viruses within a limited geographic extent [13–15]. Nevertheless, certain factors have been consistently associated with HPAI risk in domestic poultry [14, 16], including the presence of domestic livestock populations [17, 18], lower altitudes [15, 16], proximity to bodies of water [14], and travel accessibility [19].

In this study, we used evidence from previous studies [14–19] to develop a spatial framework utilizing a factor-based approach to quantify the geographic variation of animal influenza outbreak emergence potential (OEP) on a global scale. We built an OEP metric to identify geographic areas with increased potential for animal-to-human virus spillover and onward transmission. We compared the resultant risk profiles with a global index of surveillance activities for domestic poultry and swine between 2000 and 2014. The combination of spatially explicit metrics and surveillance data highlighted potentially important gaps in recent surveillance activities.

## METHODS

### Ecological Vulnerability

The OEP metric was based on the arithmetic mean of 2 equally weighted indices: (1) the ecological vulnerability index (EVI), representing the potential for animal-to-human transmission and (2) the onward transmission index (OTI), representing the potential for secondary human-to-human transmission. Different EVIs were calculated for domestic poultry and swine. For domestic poultry, the EVI incorporated 5 equally weighted indicators previously linked with avian-to-human influenza virus transmission: magnitude of co-occurrence of human and chicken populations, altitude, distance-to-water, and global distributions of extensively and intensively farmed chickens [16, 20]. Chickens were used as a proxy for domestic poultry because they were the only poultry group with available global distribution data. For swine, the EVI incorporated 5 equally weighted factors: magnitude of co-occurrence of human and swine populations, altitude, and global distributions of extensively, and intensively and semi-intensively farmed pigs [21].

Layers representing the magnitude of human and animal population co-occurrence were used to capture the density and spatial extent where both human and animals were present and where animal-to-human transmission could occur. Global layers of farming activities by management type were used because they represent contact points for increased animal-to-human transmission [22]. Distance-to-water was included in the avian-to-human transmission metric because it represents a proxy for potential interactions between wild birds and domestic poultry near open bodies of water [14, 23]. Altitude has been identified as a significant risk predictor [15, 16] and a surrogate indicator of other variables (eg, slope, land cover type). Other known factors associated with animal-to-human transmission (eg, live bird markets [24] and agricultural fairs [25]) were excluded because global distribution data were unavailable. In addition, climatic factors were omitted because their effects vary by region throughout the globe [16].

We compiled a human population density dataset using the Gridded Population of the World, version 4 (http://sedac.ciesin.columbia.edu/data/set/gpw-v4-population-density). Chicken and swine population density datasets, together with global distribution of extensive, intensive, and semi-intensive farming systems, were sourced from Livestock Geo-Wiki [26, 27]. Altitude data were gathered from WorldClim, version 1 [28], and a distance-to-water dataset was obtained as previously described [29].

### Onward Transmission

Our OTI incorporated 5 equally weighted indicators promoting secondary human-to-human transmission: human population density, travel accessibility (a measure of travel time to major cities, see [30]), national gross domestic product (GDP) per capita, national healthcare expenditure per capita, and national human seasonal influenza surveillance data (calculated from the mean number of samples submitted per country to the World Health Organization’s FluNET database [www.who.int/flunet/] from 2011 to 2015, divided by the country’s human population density). Datasets of national GDP and healthcare expenditure per capita were obtained from the International Monetary Fund (http://www.imf.org/external/pubs/ft/weo/2016/01/weodata/index.aspx) and INFORM (http://www.inform-index.org/Results-and-data/INFORM-2015-Results-and-data), respectively.

### Outbreak Emergence Potential Metric

Global datasets for each of the factors described above were imported into ArcGIS, version 10.2 (ESRI Inc., Redlands, CA) and converted into raster format for preprocessing and data management. All factors were assembled at a spatial resolution of 0.08333 decimal degrees (approximately 10 km^2^ at the equator) and resampled as necessary using bilinear interpolation corresponding to the coarsest resolution of input available for analysis. All datasets (except human seasonal influenza surveillance) were log_10_(x + 1) transformed to approximate normal distributions to reduce the skewness of indicator data and to maintain the proportion of difference in the indicators’ real values [31]. A Box-Cox transformation was applied to the human seasonal influenza surveillance dataset because its distribution was strongly zero-inflated. A lambda value of 0.2906 was used to transform the human seasonal surveillance data into an approximate normal distribution using the “forecast” package in R.

The development of EVI and OTI required the rescaling of raw indicator data values [32]. Minimum-maximum normalization was applied to each indicator to preserve the rescaling factor and exclude the distortion effect caused by outliers within the dataset. All indicator raster layers were transformed to values between 0.0 and 10.0, where higher values corresponded to increased risk. Any indicator factor that negatively contributed to the development of either index was inversely rescaled (eg, low altitude was associated with increased risk). Uniqueness among factors within each composite index was identified by both correlation and principal component analysis, and redundant factors (ie, national GDP) were removed. Two separate EVIs were developed for domestic poultry and swine by calculating the arithmetic mean of all variables associated with animal-to-human transmission. We calculated a single OTI by using the arithmetic mean of all factors associated with increased human-to-human transmission. Two separate OEP metrics were developed for domestic poultry and swine by calculating the arithmetic mean of their respective EVI and the OTI. Each factor was equally weighted because the trade-offs between indicator dimensions were unknown and the assignment of differential weights could not be justified (see Supplementary Data 1 for details).

### Ecological Vulnerability Index Validation

Human infections with swine- and avian-derived influenza viruses between January 2000 and December 2014 were extracted from the Food and Agricultural Organization (FAO) EMPRES-i Global Animal Disease Information System database (http://empres-i.fao.org/eipws3g/). To identify additional records, we performed a systematic analysis of all influenza virus hemagglutinin sequences available in the Global Initiative on Sharing All Influenza Data (GISAID) platform (platform.gisaid.org) designated as being of human origin. For each sequence, we collected the top 50 search hits that included a year stamp before the query sample year and performed a similarity analysis against all virus sequences regardless of host species origin. For each query, we parsed the host information from these top 50 hits and classified the virus to be of recent zoonotic origin if the closest search hits were from animal hosts [33]. In total, our validation dataset contained 742 human infections of avian origin and 273 human infections of swine origin with location information to the state/province level. There were >1000 other documented cases of human infections with animal influenza viruses, but these only contained country-level location data and were thus excluded (for full details, see Supplementary Data 1).

To investigate the robustness of the EVI, we compared the distribution of reported human infections with avian or swine influenza viruses against estimated maximum EVI scores at the state/province level, because the travel history of most human cases was unknown. In contrast, the OEP metric could not be validated because comprehensive data on sustained secondary human-to-human transmission of influenza virus spillover events were unavailable.

### Global Surveillance Metric

We compiled a database of 359105 reported animal influenza surveillance activities from January 2000 to December 2014, incorporating viral genetic sequences, records of influenza-positive animals, and data from documented surveillance programs. Animal influenza sequences were obtained from GISAID. Records of influenza-positive animals were derived from the FAO EMPRES-i database. Data from surveillance studies on avian and nonhuman mammalian species were acquired from the Influenza Research Database (www.fludb.org). We performed 2 systematic literature reviews, screening 10327 published studies indexed in PubMed and following recognized guidelines [34], for both poultry and swine influenza virus surveillance (see Supplementary Data 2). To identify national surveillance initiatives that may not have shared their findings through any of the above mechanisms, we performed a supplemental web search for information from national registrars and reports.

We collated and georeferenced all records to the finest level of spatial resolution available (ie, points for exact locations and polygons for administrative units). Administrative boundaries were obtained from the FAO in the form of Global Administrative Unit Layers (GAUL), where GAUL_0 indicates national boundaries, GAUL_1 indicates state/province boundaries, and GAUL_2 indicates county/district boundaries [35]. For viral sequences from GISAID, information from the sequence name itself often added geographic details beyond the accompanying sequence metadata, which were used to improve georeferencing. The resulting database was used to develop the surveillance metric by assigning a score of 1 for each calendar year in which evidence for any surveillance activity was reported for each geographic area. Scores for the surveillance metric ranged from 0 (no surveillance) to 15 (surveillance for every year from 2000 to 2014). Years of surveillance did not need to be consecutive, and all records were treated equally to reduce inherent reporting biases due to surveillance activities not producing any positive reports (eg, a single record of animal influenza from a country would receive the same surveillance score as 100 records from a different country collected within the same year). Maps were produced to illustrate the spatial extent of influenza surveillance in domestic poultry and swine at each of the 3 GAUL between the January 2000 and December 2014 (see Supplementary Data 2 for further details on methodologies and databases).

## RESULTS

### Outbreak Emergence Potential Metric

**Figure 1.**
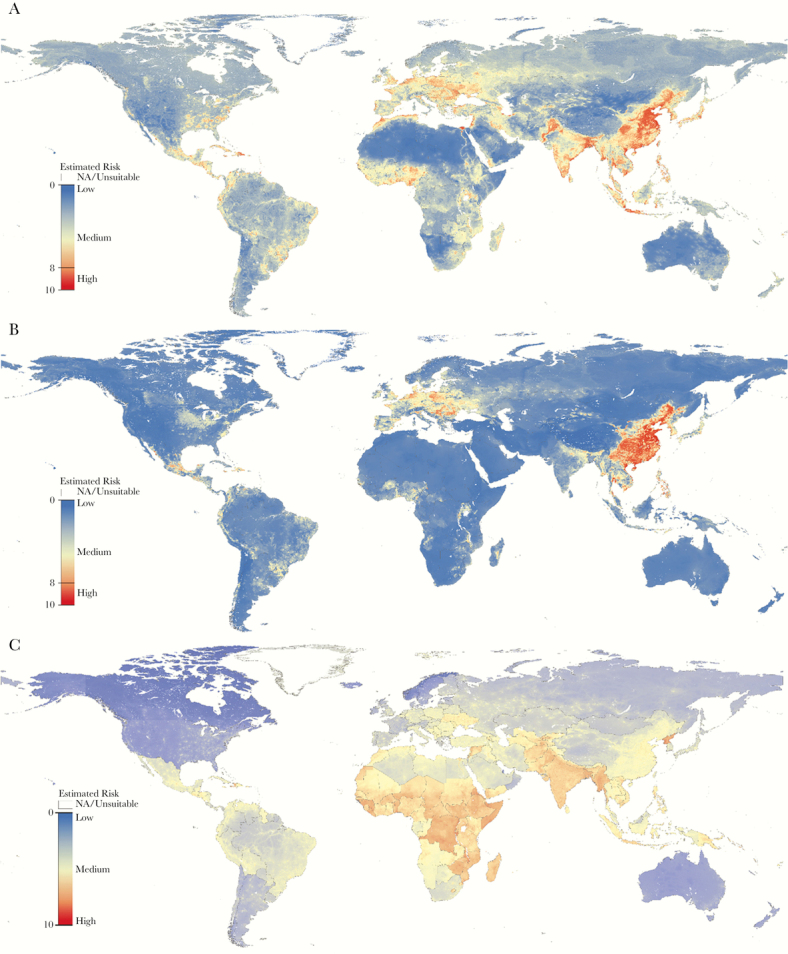
Variation in the estimated ecological vulnerability index (EVI) and onward transmission index (OTI) for animal influenza virus transmission. Chicken-to-human EVI (A), swine-to-human EVI (B), and secondary human-to-human OTI (C) transmission risk at 10 km^2^ resolution is displayed on a gradient from blue (ie, low risk) to red (ie, high risk).

Mapping of the EVI illustrated areas of potential avian-to-human influenza virus spillover, with hotspots identified in regions of South and Southeast Asia, Eastern Europe, the Southeastern United States, Central America, West Africa, the Maghreb, and the Nile River Delta. Moreover, considerable foci for potential swine-to-human virus spillover events were concentrated in Southeast Asia, Eastern Europe, and Central Mexico. Mapping of the OTI displayed areas likely to promote secondary human-to-human transmission, largely in Central Asia, Eastern Europe, and sub-Saharan Africa (Figure 1).

**Figure 2.**
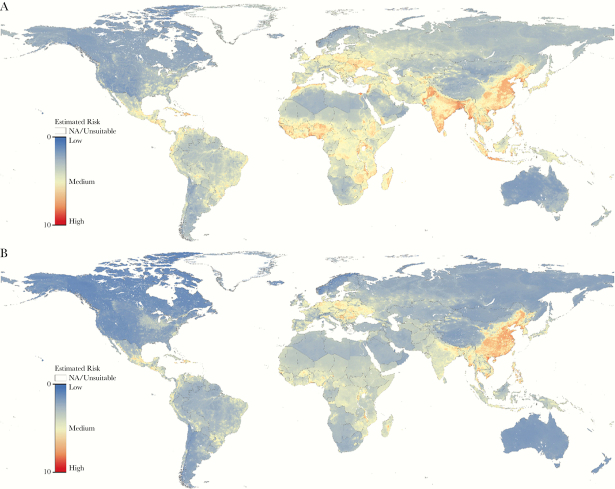
Variation in the estimated outbreak emergence potential metric for animal influenza from domestic poultry (A) and swine (B) at 10 km^2^ resolution is displayed on a gradient from blue (ie, low risk) to red (ie, high risk).

The OEP metric displayed considerable global variation. Regions with elevated EVI scores for domestic poultry and/or swine resulted in both higher and lower OEP scores (eg, West Africa and the Southeastern United States, respectively) due to their OTI ranking. Overall, regions either with (eg, Southeast Asia) and without (eg, Eastern Europe and West Africa) prior history of human infection outbreaks for avian and/or swine influenza viruses were ranked highly in our OEP metric (Figure 2).

### Ecological Vulnerability Index Validation

**Figure 3.**
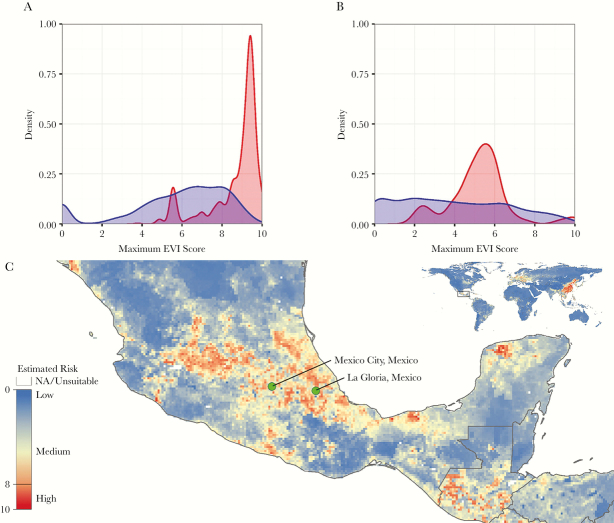
Global maximum ecological vulnerability index (EVI) scores at the state/province level for chicken-to-human (A) and swine-to-human (B) risk of animal influenza virus transmission. Estimated EVI scores are displayed in blue, whereas the number of human infections with influenza viruses of animal origin are in red. Swine-to-human EVI showing the regions of Mexico City and La Gloria, Mexico, as sites at increased potential for zoonotic transmission (C). The map has a resolution of 10 km^2^ and is displayed on a gradient from blue (ie, low risk) to red (ie, high risk).

Our EVI highlighted substantial geographic variability in the potential for influenza virus spillover events from domestic poultry and swine populations. We compared the estimated EVI at the state/province level to the number of human infections with animal influenza viruses from that state/province. For human infections with influenza viruses of avian origin, 79.91% of the reported cases (593 of 742) came from states/provinces with maximum EVI scores ≥8 of 10 (10 being the highest potential for animal-to-human transmission [Figure 3A]). Of the remaining 149 human cases observed in areas with maximum EVI scores <8, 77 were from locations adjacent to areas with maximum EVI scores ≥9, whereas 72 were adjacent to areas with maximum EVI scores ≥8. For example, in Jakarta, Indonesia, 47 human cases of infection with avian influenza virus were reported. Although Jakarta had a maximum EVI score of 5.6, the Indonesian provinces bordering it have maximum EVI scores of 9.6 (Jawa Barat) and 9.5 (Banten).

For human infections with influenza viruses of swine origin, comparisons were not informative because 262 of 273 reported cases in our dataset came from the United States where maximum EVI scores ranged from 2.0 to 6.7. In contrast, many of the highest EVI scores (≥9) came from provinces in China, whereas only 3 cases were observed there. The remaining 8 human cases were observed in the states/provinces of Canada, Hong Kong, Kazakhstan, and Spain with maximum EVI scores <8 (Figure 3B). It is interesting to note that the EVI for swine-to-human transmission risk (Figure 1B) was high in central Mexico, including the area of La Gloria (maximum EVI score ≥8) (Figure 3C), the putative site of emergence of the 2009 “swine flu” pandemic [36]. The high EVI and moderate OTI scores of the area around La Gloria resulted in an overall OEP score, suggesting that this region had substantial potential for the emergence and spread of animal influenza viruses (Figure 2).

### Global Surveillance Metric

**Figure 4.**
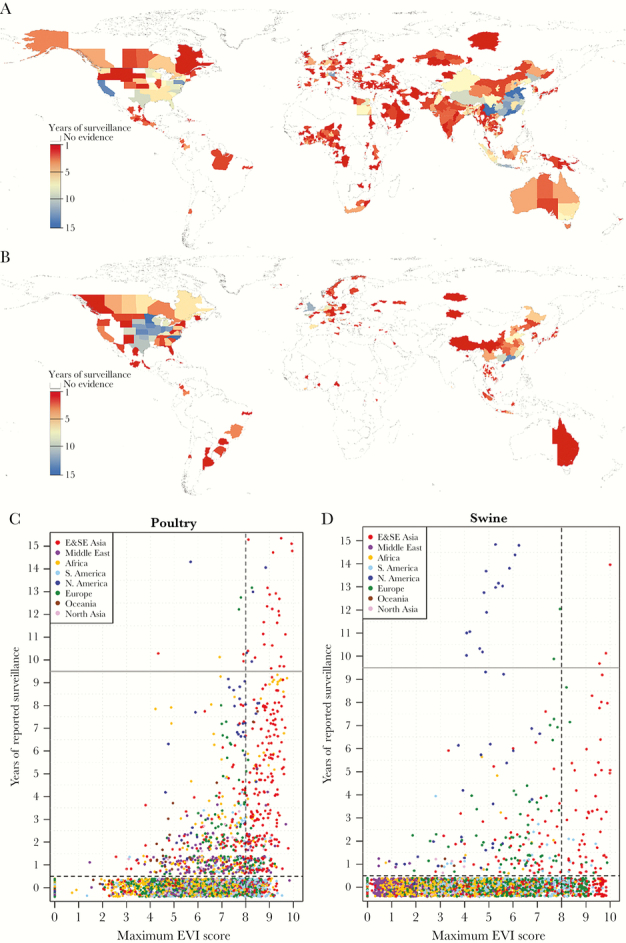
Global variation in poultry and swine influenza surveillance by state/province from 2000 to 2014. Surveillance metric results for domestic poultry (A) and swine (B), where years of surveillance are displayed on a gradient from 1 (red) to 15 (blue). Years of surveillance were compared with estimated maximum ecological vulnerability index (EVI) scores for chicken-to-human (C) and swine-to-human transmission (D).

We found substantial variability in surveillance activities over space and time despite applying conservative assumptions in the metric development. For most countries, we found evidence of animal influenza surveillance at a national level, although it varied substantially each year. Evidence for surveillance decreased markedly when filtered by data available at subnational spatial resolution (ie, GAUL_1 and GAUL_2). Data aggregation to the GAUL_1 level (state/province) showed that the majority of domestic poultry surveillance activities were concentrated in areas at high risk for avian-to-human transmission of influenza viruses (Figure 4A). However, 49.01% of the states/provinces at high risk (maximum EVI ≥8) lacked any evidence of surveillance in the last 15 years (Figure 4C). Furthermore, 25 of the 124 countries reporting surveillance in domestic poultry had data spanning longer than 10 years, whereas 42 countries reported surveillance for ≤3 years. Countries with high-risk areas (maximum EVI ≥8), such as Belarus, Brazil, Honduras, and the Philippines, had ≤3 years of national surveillance.

Little overlap was found between areas at elevated risk (maximum EVI ≥8) for swine-to-human transmission of influenza viruses and evidence of surveillance. Most states/provinces with multiple consecutive years of surveillance activities were in the United States, which had maximum EVI scores <8. In areas at higher risk of zoonotic transmission, only southeastern China and Hong Kong had multiple consecutive years of surveillance. In contrast, the remaining states/provinces at higher risk had intermittent surveillance activities despite the high numbers of swine present (Figure 4B). Of the states/provinces identified as high risk (maximum EVI ≥8), 78.43% lacked any surveillance activities in our dataset (Figure 4D). Furthermore, 15 of the 67 countries reporting surveillance in swine had data spanning longer than 10 years, whereas 32 reported surveillance for ≤3 years. Countries with high-risk areas, such as Guatemala, Philippines, Serbia, and Romania, had ≤3 years of national surveillance. Details on data analysis at the GAUL_0 and GAUL_2 levels are provided in Supplementary Data 2.

## DISCUSSION

Our results indicate (1) substantial geographic variation in global virus spillover potential from domestic poultry and swine populations to humans and (2) substantial heterogeneity in onward transmission risk. With limited funding allotted to active surveillance, our global framework could identify priority areas to make the best use of available resources. A variety of countries in Central America (eg, Guatemala, Honduras), Eastern Europe (eg, Romania, Serbia), West Africa, and Southeast Asia scored highly in their EVI, OTI, and OEP, suggesting that they are key areas for future investments in surveillance and pandemic preparedness. It is interesting to note that the area of La Gloria, Mexico, scored highly in the swine EVI and moderately in the OTI, displaying elevated OEP conditions. This finding provided support for our OEP metric’s ability to indicate situational awareness of elevated risk conditions and emphasized the need for more surveillance in areas with similarly high scores.

We found that the majority of surveillance efforts in domestic poultry were focused in areas where avian-to-human virus spillover risks were high and previous human infections had been documented (eg, China, Hong Kong, Thailand). However, we also found areas at equally high risk but without any evidence of surveillance in poultry (eg, Belarus, Honduras). For swine, the greatest concentration of high-risk areas for swine-to-human transmission was in Southeast Asia, whereas similarly high-risk areas were found in Eastern Europe and Central America.

Our study has several limitations, such as the variety of ecological factors, which could not be included in our metrics on a global scale. For example, live bird markets and agricultural fairs are important settings for the transmission of influenza viruses between animals and humans [24, 25]. However, global data on domestic animal movement and trade are unavailable. The generation of such datasets would require enormous time and financial commitments. Nevertheless, this information could be integrated into the modeling framework used here, improving the risk assessment of animal influenza outbreak and spillover potential.

A second limitation is the uncertainty surrounding the factors facilitating cross-species transmission and contributing to onward pathogen spread. Our models could be improved with more information on the ecology of cross-species transmission. Likewise, our approach could be used as a framework to quantify these ecological variables. For example, the EVI validation for avian influenza virus spillover suggests that this metric can be used to identify areas with increased potential for zoonotic transmission.

A third limitation is that reporting of human infections with animal influenza viruses in areas with low EVI scores suggests a potential detection/recording bias, or this may be due to other risk factors not accounted for in our model. Although very few human infections with influenza viruses of swine origin were reported in states/provinces with EVI scores ≥8, this pattern is likely to be associated with detection biases. Human infections with swine influenza viruses are rarely severe [37], and most reported cases are from countries with substantial human influenza surveillance programs [38]. By contrast, human infections with avian influenza viruses are often fatal and more likely to be detected and reported.

Our study, derived solely from publicly accessible data, provides a new illustration of the temporal and geographical variation in global animal influenza surveillance activities. We attempted to be comprehensive in identifying reported surveillance activities, but there are undoubtedly some efforts that we might have missed either because they were not reported publicly or because the reports fell outside of our search strategies. Nevertheless, there is a clear need to increase levels of surveillance in key locations worldwide.

Our approach to standardizing and georeferencing each surveillance record highlights the necessity of a unified global influenza virus surveillance data management plan where active and passive surveillance components are linked and their results made readily available to the international community [5]. Currently, animal influenza virus data are caught in the void between international organizations responsible for animal welfare and trade and those responsible for human health. The absence of a single authoritative organization responsible for surveillance inhibits a global response system for influenza virus tracking. In addition, the lack of data standardization and quality control impedes the effective use of the relatively limited amount of information that enters the public domain. Similar shortcomings were identified during a recent Ebola outbreak, catalyzing a call for global public health agencies to coordinate an improved epidemiological data management system for disease surveillance [39, 40].

## CONCLUSIONS

Expanded surveillance in both domestic poultry and swine is imperative to identify novel strains of potential importance and to understand the factors driving cross-species virus transmission. Our global framework highlights priority areas where improved influenza outbreak prevention and mitigation strategies are paramount, enabling national and international agencies to evaluate prepandemic preparedness needs and target surveillance resources.

## Supplementary Data

Supplementary materials are available at *Open Forum Infectious Diseases* online. Consisting of data provided by the authors to benefit the reader, the posted materials are not copyedited and are the sole responsibility of the authors, so questions or comments should be addressed to the corresponding author.

ofy318_suppl_supplementary_data1

ofy318_suppl_supplementary_data1_figure_1

ofy318_suppl_supplementary_data1_figure_2

ofy318_suppl_supplementary_data1_figure_3

ofy318_suppl_supplementary_data1_figure_4

ofy318_suppl_supplementary_data1_figure_5

ofy318_suppl_supplementary_data1_figure_6

ofy318_suppl_supplementary_data1_figure_7

ofy318_suppl_supplementary_data2

ofy318_suppl_supplementary_data2_figure_1

ofy318_suppl_supplementary_data2_figure_2

ofy318_suppl_supplementary_table_1

ofy318_suppl_supplementary_table_2

ofy318_suppl_supplementary_table_4

ofy318_suppl_supplementary_table_3
