# Supplementary material for: The Geographic Variation of Surveillance and Zoonotic Spillover Potential of Influenza Viruses in Domestic Poultry and Swine
Source: Open Forum Infect Dis. 2018 Nov 27;5(12):ofy318. doi: 10.1093/ofid/ofy318 (PMC6309522; doi:10.1093/ofid/ofy318)
Supplement: ofy318_suppl_supplementary_data1 [file ofy318_suppl_supplementary_data1.docx]

**SUPPLEMENTARY DATA 1**

**The geographic variation of surveillance and zoonotic spillover potential of influenza viruses in domestic poultry and swine**

Kathryn A. Berger, David M. Pigott, Francesca Tomlinson, David Godding, Sebastian Maurer-Stroh, Biruhalem Taye, Fernanda L. Sirota, Alvin Han, Raphael T.C. Lee, Vithiagaran Gunalan, Frank Eisenhaber, Simon I. Hay, Colin A. Russell

**SUPPLEMENTARY METHODS**

**Geospatial datasets and indicator selection**

A general workflow diagram providing an overall view of model development and associated analyses is provided in Supplementary Data 1 Figure 1. A description the variables selected for both index and metric development, as well as the methodologies used to derive and calculate them, can be found below.

*Population densities at the human-animal interface*. Human cases of avian influenza typically originate from domestic poultry populations due to frequent and intense exposure in both industrially reared and extensive (or “backyard”) settings [1]. The spread of HPAI H5N1 and H7N9 from animals to humans has been found to be primarily influenced by human activities related to domestic poultry production and trade [2,3]. In addition to domestic poultry, swine are also a reservoir of influenza viruses with frequent virus transmission to and from humans.

Increased contact between humans and animals increases the risk animal-to-human virus transmission. For populations occupying the same ecologically-relevant geographic area, increases in human and animal population density are likely to elevate the risk of cross-species virus transmission. However, the intensity of contact between animal and human populations is likely to be mediated by other exogenous factors such as agricultural practices (high-biosecurity commercial settings versus backyard livestock systems) and natural ecological settings.

To capture the density and extent of population overlap we assembled a population density layer to quantify areas where both humans and animals were present. Areas lacking poultry and/or humans, as well as swine and/or humans, were assumed to have no risk of animal-to-human transmission from either domestic species. A combined measure of co-occurrence for both human and animal populations was developed by selecting those areas where either human or animal population density was greater than zero. Those selected areas were then multiplied against each other (e.g., [global human population density >0] * [chicken population density >0]) and scaled from 0.0 to 10.0. In this way, areas where only one value was present would not be assigned an increased risk value. Areas where neither human nor animal population distributions existed were masked out as areas of unsuitability (see [4-6] as sources for data layers of human and animal population densities).

*Distance-to-water*. Distance-to-water has been identified as a significant factor for increased HPAI risk in domestic poultry settings [7-10]. We included this layer in the model of avian-to-human transmission due to the increased potential interaction between wild birds and domestic poultry near open bodies of water. This component was excluded from the swine-to-human model as it lacked documented biological relevance in transmission dynamics (see [11] as source for the distance-to-water layer).

*Altitude*. A negative association between altitude and HPAI risk has been identified by the literature, suggesting a ‘protective effect’ of higher altitudes against HPAI incidence [12]. Defined as elevation above sea level in meters (m), this raster layer ranged from -431 m (below sea level) to 8,233 m above sea level [13].

*Global accessibility***.** Defined as the travel time (in minutes) to reach a population center >50,000 people where modes of travel are both land- and water-based [14]. This parameter has been found to have a positive association with increased risk for HPAI [15] and would likely affect the rate of onward spread of human-adapted influenza virus.

*Pandemic preparedness*. Direct measures of pandemic preparedness (or the ability to control the spread of a newly emerged virus) were not available on a global scale. However, the ability of a country to dedicate financial resources to controlling the spread of a newly emerged virus is likely to be associated with increased probability of early detection and decreased risk of onwards transmission. Again, direct measures of such resources and willingness are rare, so we considered three proxies:

1. *Gross domestic product (GDP).* For GDP, per capita estimates in units of purchasing power parity (PPP) were derived from the International Monetary Fund (IMF) World Economic Outlook (in USD) and used to describe the economic performance at the national level (April 2016 edition, http://www.imf.org/external/pubs/ft/weo/2016/01/weodata/index.aspx); a mean value was calculated from the year 2010 to 2016. Values were reported in terms of PPP in order to provide a more accurate measure of what can be bought using income in the national currency, providing a better metric for cross-country comparison. Where national values were not available through the IMF, supplemental resources were consulted (CIA World Factbook [https://www.cia.gov/library/publications/the-world-factbook/rankorder/2004rank.html]; the World Bank [http://data.worldbank.org/indicator/NY.GDP.PCAP.PP.CD#]).
2. *Healthcare expenditure.* National spending on healthcare infrastructure has been identified as a significant component in predicting the international spread of zoonotic viruses [16]. The inclusion of this socio-economic variable captures an individual’s ability to seek treatment and the country’s capacity for surveillance and identification of pathogens [16]. These data, obtained from INFORM (a global open-source risk assessment for humanitarian crises and disasters; http://www.inform-index.org/Results-and-data/INFORM-2015-Results-and-data), were also described in units of PPP and reported the amount of money (in USD) spent by each nation on healthcare. Values documented for mid-2015 were used for the development of this metric. Where national healthcare expenditure data were unavailable from the INFORM index table, values were derived from The World Bank (http://data.worldbank.org/indicator/SH.XPD.PCAP). For all other missing values (the majority of which were small islands) a constant value of $10,634.30 was assigned. This was calculated as the average between the value assigned for Caribbean small states (USD 15,587.20) and the value assigned for Pacific Island small states (USD 5,681.40), derived from the World Bank.
3. *Human seasonal influenza surveillance.* Early detection of a newly emerged virus is likely to rely on existing surveillance infrastructure for respiratory pathogens. Human seasonal influenza viruses are among the most monitored pathogens in the world with an extensive global reporting infrastructure administered by the World Health Organisation (WHO). To estimate the amount of influenza surveillance by country, we calculated a per capita surveillance rate as the mean number of specimens processed per country per year from 2011 to 2015 (as reported to WHO, available via www.who.int/flunet/) and dividing that mean by the human population size of that country in 2014. For some countries, the number of specimens processed was not reported. For these countries, we used the total number of positive samples divided by the global mean percent positivity rate to generate a rough estimate of number of samples processed. For countries with no reports to www.who.int/flunet/ during the period 2011-2015, we assumed a constant near-null value for surveillance.

Further metadata on all indicator variables can be found in Supplementary Data Table 1 (for ecological vulnerability) and Supplementary Data Table 2 (for onwards transmission). While we used the most contemporary datasets available for metrics development, it should be observed that there was a range of 10 years between the oldest and newest datasets. Maps of all indicator layers used in the ecological vulnerability index (EVI) development can be found in Supplementary Data 1 Figures 2-3. In the absence of additional information for the development of the swine-specific EVI, we opted to use the same basic formulation as for avian-to-human transmission, except the distance-to-water dataset. This is because the ecological factors associated with swine-to-human influenza virus transmission are much less well studied than avian-to-human factors. Maps of all indicator datasets used in the onward transmission index (OTI) development are illustrated in Supplementary Data 1 Figure 4.

**Imputation of missing data**

Missing data can distort the real value of a composite indicator. Where values are missing, the metric aggregation process fails as a tool to compensate a deficit in one component by surplus in another [17]. Missing data cannot be completely avoided, especially at a global scale. Imputation of missing national socio-economic values (GDP and healthcare expenditure) were determined using constant values described as the average of similar states. In cases where countries are facing internal conflict (e.g., Syria) and data were unattainable and/or out-of-date, the resultant composite cross-species transmission index should be interpreted with caution. See Online Table 1 for imputation of all national level indicator data, by country and source.

*GDP.* Where IMF data were unavailable, constant values were provided from the CIA Factbook with the date of estimation. When neither of these resources could provide estimated values, additional sources were used to impute values for individual countries (Supplementary Data 1 Table 3).

*Healthcare expenditure.* Healthcare expenditure values were sourced from the INFORM-Index 2016 spreadsheet, made available from http://www.inform-index.org/Results/Global, accessed 08/01/2016 (mid-2015 values). Where data from the INFORM-Index table were not provided, values were derived from the World Bank (http://data.worldbank.org/indicator/SH.XPD.PCAP, accessed 08/01/2016). This was the same methodology as used above for GDP of small states and low-income areas where constant values derived from World Bank were assigned.

**Metrics Development**

A total of three indices were developed: 1) an EVI for virus transmission from domestic poultry to humans; 2) an EVI for virus transmission from swine to humans; and 3) an OTI for secondary human-to-human spread. The combination of the EVIs and OTI resulted in our final outbreak emergence potential (OEP) metric. This section provides additional methods and procedures as to how these models were developed.

Prior to the development of each composite index, raw indicator data values required rescaling [see 18]. Minimum-maximum normalization was used to rescale each indicator variable, to preserve each variable re-scaling factor, and to exclude the distortion effected caused by outliers. This method is normally applied in deductive index designs, as employed in this case, and is preferred when a dataset contains extreme or outlier values [19]. Once minimum and maximum values were identified, all indicator variables were transformed on a min-max scale (between 0.0 and 10.0), where higher values indicated increased risk. Boxplots were used to visualize, define minimum and maximum distribution bounds and to identify outliers. All indicator variables were rescaled using the Equation_1 below:

$x_{i,norm}^{j}=\frac{x_{i}^{j}-x_{i,min}}{x_{i,max}-x_{i,min}} x 10$ Equation_1 [17]

Where $x_{i}^{j}$is the data point for the j^th^ country from the i^th^ indicator’s dataset, $x_{i,min}$is the minimum value for the i^th^ indicator’s dataset, $x_{i,max}$is the maximum value for the i^th^ indicator’s dataset, and $x_{i,norm}^{j}$ is the normalized data point for the j^th^ country from the i^th^ indicator’s dataset. With the notion that higher values indicated higher risk, any indicator that would negatively contribute to the development of the metric was inversely rescaled. For example, an increase in money spent on national healthcare systems would be expected to decrease the potential for onward transmission as improved surveillance and healthcare infrastructure would facilitate early identification of human infections with novel influenza viruses. Based on this logic the indicator variable for national healthcare expenditure would be inversely rescaled using Equation_2. As a result, the following layers were inversely rescaled: ‘Accessibility – Travel time to major cities’; ‘altitude’; ‘distance-to-water’; ‘FluNet human influenza surveillance’; ‘national GDP’; and ‘national healthcare expenditure.’

$x_{i,norm,inv}^{j}=10- x_{i,norm}^{j}$ Equation_2 [17]

Where $x_{i,norm}^{j}$ is the normalized data point for the j^th^ country from the i^th^ indicator’s dataset and $x_{i,norm,inv}^{j}$ is the normalized data point for the j^th^ country from the i^th^ indicator’s dataset inversed.

A correlation table was used to identify indicators that were highly associated with each other, and the principal component analysis was used to identify those variables that could be safely removed from composite index construction without biasing the resulting risk estimate. As a result of these analyses, national GDP was removed from the OTI as it was highly correlated with healthcare expenditure (*r=0.92928*), but contributed less to the final composite metric. Equal weighting was applied as the trade-offs between indicator variable are not fully understood and therefore assignment of differential weights could not be justified. Composite index values for both the ecological vulnerability and onward transmission were aggregated by calculating the arithmetic mean of all indicators.

Our OEP metric was developed as the result of the arithmetic mean of both the EVI and OTI indices, which ranged in value from 0.0 to 10.0, where higher values suggest increased ecological vulnerability to animal-to-human disease dynamics and greater potential for rapid onwards transmission.

**EVI validation**

To ensure that we included as many human infections with animal influenza viruses as possible, and to complement the EMPRES-i database of reported human infections, we systematically analyzed all publicly available human influenza virus sequences to identify human infections. To obtain a comprehensive set of publicly available genetic sequences of influenza viruses collected from humans, we downloaded all influenza virus hemagglutinin protein sequences in GISAID (http://platform.gisaid.org) with minimum length of 550 residues and host species listed as “human” (n=29,996 on October 07, 2015). Sequences of A(H1N1)pdm09 lineage viruses were excluded as their zoonotic origin is already well established. For each virus sequence, we ran Tachyon search tool [20] to compare a specific influenza virus sequence with all other influenza virus sequences in the large non-redundant database that is pre-indexed and connected to the Tachyon web service (http://tachyon.bii.a-star.edu.sg/).

Briefly, the Tachyon search tool rapidly identifies similar protein sequences by searching a reduced version of a given protein sequence database. Searched sequence space is initially reduced by associating each entry in the database with five representative pentapeptides. The Tachyon search then proceeds in two steps: 1) it returns database entries sharing a defined number of pentapeptides with the query; and 2) hits returned by the pentapeptide search are subjected to a more detailed search over the full length sequences in order to evaluate the significance of each hit.

For each virus sequence we collected the top 50 search hits that have a year stamp before the query sample year. This was necessary to ensure that we only considered viruses from preceding years as potential ancestors. For each query we parsed the host information from these top 50 hits and classified the virus to be of recent zoonotic origin if a critical number of closest search hits were from animal hosts. We tested thresholds of 75%, 50%, and 25% of animal hits as well as further screened all those with at least one hit of animal origin to assess all potential events. The computational algorithm was able to evaluate the origins of all viruses labelled as “human” in GISAID in ~12 hours, followed by further manual curation. The automatically detected zoonotic events from human sequences were curated with the main criterion that the identified events should be likely zoonotic (and therefore excluding human-to-human transmission like all A(H1N1)pdm09-lineage viruses since 2009 except one reference introduction), and with removal of redundant counts from alternative passages/recombinants of the same isolate (e.g., A/Anhui/1/2013(H7N9)).

In total, the algorithm and subsequent filtering returned 832 human-isolated viruses with at least one animal-hosted virus in the top hits from preceding years that we classified as zoonotic cases (Online Table 2A). At thresholds of 75%, 50%, and 25% of hits coming from animal origin, the number of viruses automatically flagged as zoonotic were 638, 796, and 821 respectively. Seven of the 11 cases below 25% are made up of early or remote swine-origin sequences with few closely related sequences available. The remaining 4 cases were H5N1 avian influenza cases from a 2007 outbreak in Indonesia where mostly human cases have been sequenced although multiple avian-to-human rather than human-to-human transmissions should be suspected.

Among the 832 zoonotic cases with sequences, 75% were from avian origin. By host and subtype, the majority were avian H5N1 (47%), followed by avian H7N9 (20%), swine H3N2 (19%), avian H7N7 (6%), swine H1N1 (5%), avian H9N2 (1%), and a remaining 1.5% formed by swine H1N2, avian H5N6, avian H10N8, avian H7N3, avian H6N1, and avian H7N2. We observed a sharp increase in sequences available for zoonotic events from 2003 onwards, which is dominated by the avian influenza viruses and a peak from swine H3N2 in 2012.

When viewing the zoonotic cases in the context of their geographic location, a clear pattern appears showing that reported swine-origin zoonotic infections had a strong focus in the US Midwest (Supplementary Data 1 Figure 6). Conversely, reported avian-origin zoonotic infections demonstrated a broader spread of origins, but with clear hotspots in southern China forming a belt into Southeast Asia and Indonesia, with a mix of H5, H7, and H9 types (Supplementary Data 1 Figure 7). This large region is also the source of human infections with several unique subtypes such as H5N6, H6N1, and H10N8. Additionally, repeated cases of H5N1 came from Egypt and the Middle East as well as H7 types in Europe.

Next, we compared the systematically derived list of zoonotic cases with previously published lists including the 2015 WHO report for human H5N1 cases [21], three comprehensive reviews [22-24], and the human infections with animal influenza viruses included in EMPRES-i. Importantly, it should be noted that in most cases only year, country, host, and subtype were listed and therefore we had to compare our list at the same level of reduced detail. Thus many of the sequences identified through our searches could not be included in the map validation. The number of reported zoonotic events in these reviews were 67, 19, 35, and 68 (Online Table 2B) with surprisingly large complementarity leading to a non-redundant total of 142 zoonotic events (Online Table 2C). Although only 96 (67.6%) of the previously reported events matched cases in our systematic list, 35 of the 46 events not returned in our sequence-based search simply did not have any sequence information in public databases and from the remaining 11, nine had sequences too short to be considered in our approach (<550 amino acids), one was an exceptional case of a reassortant seasonal virus [25], while the last one had a mismatch of the geographic location in the strain name compared to the described zoonotic event (Online Table 2C). In summary, there were a considerable number of zoonotic events reported in these reviews without sequences in public databases but for cases with good quality sequences available, our approach identified all of them. However, our counts underestimate the number of actual cases by ignoring multiple cases from the same location in the same year. For example, there can be multiple independent transmissions in a year within the same location, as has been the case for avian H5N1 viruses in China, Indonesia, Egypt, and Cambodia or also different clades within a subtype in one country (e.g., Vietnam).

Based on all of the evidence for human infections with swine or avian influenza described above, and filtered for reports that had spatial information resolved to at least the state/province level, we validated the estimated ecological vulnerability distribution maps against 742 avian-origin human infections and 273 swine-origin human infections (see Online Tables 3 and 4, respectively). As the spatial quality and travel history of many of the geo-positioned human cases of animal-origin influenza virus infection were unknown, the maximum ecological risk score per administrative unit (GAUL_1) was used to validate against the total number of cumulative cases in the same administrative unit area (see Figure 3A and 3B in main text). The mean EVI score was also investigated (Supplementary Data 1 Figure 5) in order test the sensitivity of our validation analysis. Given the unknown travel history of each patient, the location of where one might have acquired the animal-borne virus is difficult to determine within the georeferenced state or province. In this way, the maximum EVI score may be a more reasonable estimate of disease emergence potential; however, at least for domestic poultry, the trends are similar using the mean value. For swine, as stated in the main text, validation is less informative as its pattern is more likely to be associated with detection biases.

**Supplementary Data 1 Table 1.** Indicator variables used in the development of the ecological vulnerability index (EVI).

| Layer | Year | Scale | Reference | Note | Species |  |
| --- | --- | --- | --- | --- | --- | --- |
| Chicken (population density) | 2006 | 30 arc seconds* | 5 | Merged with human population density layer for combined indicator layer | Domestic poultry | |
| Chicken Extensive (Farming) Distribution | 2015 | 5 minutes† | 6 |  | Domestic poultry | |
| Chicken Intensive (Farming) Distribution | 2015 | 5 minutes | 6 |  | Domestic poultry | |
| Pigs (population density) | 2006 | 30 arc seconds | 5 | Merged with human population density layer for combined indicator layer | Swine | |
| Pig Extensive (Farming) Distribution | 2016 | 5 minutes | 6 |  | Swine | |
| Pig Semi-Intensive (Farming) Distribution | 2015 | 5 minutes | 6 |  | Swine | |
| Pig Intensive (Farming) Distribution | 2015 | 5 minutes | 6 |  | Swine | |
| Distance to Water | 2011 | 30 arc seconds | 11 |  | Domestic poultry | |
| Altitude | 2005 | 30 arc seconds | 13 |  | Domestic  poultry; Swine | |

*30 arc seconds (0.008333 decimal degrees, or approximately 1x1km at the equator).

†5 minutes (0.083333 decimal degrees, or approximately 10x10km at the equator).

**Supplementary Data 1 Table 2.** Indicator variables used in the development of our onward transmission index (OTI).

| Layer | Year | Scale | Reference |
| --- | --- | --- | --- |
| Gridded population of the world | 2010 | 30 arc seconds* | [4](http://www.ciesin.columbia.edu/data/gpw-v4/) |
| INFORM mid-2015 healthcare Expenditure | mid-2015 | National | <http://www.inform-index.org/Results-and-data/INFORM-2015-Results-and-data> |
| Gross domestic product based on purchasing-power-parity (PPP) per capita GDP | 2016 | National | <http://www.imf.org/external/pubs/ft/weo/2016/01/weodata/index.aspx> |
| FluNet human seasonal influenza surveillance | 2011-2015 | National | www.who.int/flunet/ |
| Accessibility Travel Time to Major Cities (in minutes) | 2008 | 30 arc seconds | 14 |

*30 arc seconds (0.008333 decimal degrees or approximately 1 x 1 km at the equator).

**Supplementary Data 1 Table 3.** Supplemental data sources used to impute missing gross domestic product values.

| Country | Year | Reference |
| --- | --- | --- |
| French Guiana | 2003 | <http://www.factbook.org/factbook/fg.shtml> |
| Guadeloupe | 2003 | <http://www.indexmundi.com/guadeloupe/gdp_per_capita_(ppp).html> |
| Martinique | 2015 | <http://www.indexmundi.com/g/g.aspx?c=mb&v=67> |
| Netherlands Antilles | 2011 | <http://www.theodora.com/wfbcurrent/netherlands_antilles/netherlands_antilles_economy.html> |
| West Bank and Gaza Strip | 2008 | <http://data.worldbank.org/indicator/NY.GDP.PCAP.PP.CD> |

**Supplementary Data 1 Figure Legends**

**Supplementary Data 1 Figure 1.** An overall workflow diagram of ecological vulnerability index (EVI), onward transmission index (OTI) and outbreak emergence potential (OEP) metric development and associated analyses performed within this manuscript.

**Supplementary Data 1 Figure 2.** Global indicator variables used in the development of the ecological vulnerability index (EVI) for domestic poultry: (A) combined chicken and human population density, (B) intensive chicken farming distribution, (C) extensive chicken farming distribution, (D) altitude, and (E) distance to water. All layers are 10 km^2^ resolution, rescaled from 0 to 10 and displayed on a gradient from blue (i.e., low risk) to red (i.e., high risk).

**Supplementary Data 1 Figure 3.** Global indicator variables used in the development of the ecological vulnerability index (EVI) for swine: (A) combined pig and human population density, (B) intensive pig farming distribution, (C) extensive pig farming distribution, (D) semi-intensive pig farming distribution, and (E) altitude. All layers are 10 km^2^ resolution, rescaled from 0 to 10 and displayed on a gradient from blue (i.e., low risk) to red (i.e., high risk).

**Supplementary Data 1 Figure 4.** Global indicator variables used in the development of the onward transmission index (OTI) for domestic poultry: (A) human population density, (B) gross domestic product, (C) healthcare expenditure, (D) travel accessibility, and (E) human seasonal influenza surveillance. All layers are 10 km^2^ resolution, rescaled from 0 to 10 and displayed on a gradient from blue (i.e., low risk) to red (i.e., high risk).

**Supplementary Data 1 Figure 5**. Global mean ecological vulnerability index (EVI) scores at the state/province level for chicken-to-human (A) and swine-to-human (B) risk of animal influenza virus transmission. Estimated EVI scores are displayed in blue, while the number of human infections with influenza viruses of animal origin are in red.

**Supplementary Data 1 Figure 6**. Geographic distribution of human infections with swine influenza viruses detected through sequence comparisons. Circles indicate geographic location of human cases. Circle size indicates number of cases, circle color indicates virus subtype. Shading of geographic map indicates modelled swine population densities from (http://livestock.geo-wiki.org/). White = no swine, light grey = low swine population density, dark grey = high swine population density.

**Supplementary Data 1 Figure 7**. Geographic distribution of human infections with avian influenza viruses detected through sequence comparisons. Circles indicate geographic location of human cases. Circle size indicates number of cases, circle color indicates virus subtype. Shading of geographic map indicates modelled chicken population densities from (http://livestock.geo-wiki.org/). White = no chickens, light grey = low chicken population density, dark grey = high chicken population density.

**REFERENCES**

1. Krauss S, Webster RG. Avian influenza virus surveillance and wild birds: past and present. Avian Dis **2010**; 54: 394-398.
2. Normile D. Wild birds only partly to blame in spreading H5N1. Science **2006**; 312(5779):1451.
3. Yiu Lai K, Wing Yiu Ng G, Fai Wong K, Fan Ngai Hung I, Kam Fai Hong J, Fan Cheng F, et al. Human H7N9 avian influenza virus infection: a review and pandemic risk assessment. Emerg Microbes Infect **2013**; 2(8):e48.
4. Center for International Earth Science Information Network, Columbia University. Gridded population of the world, version 4 (GWPv4): population density. Palisades, (NY): NASA Socioeconomic Data and Applications Center (SEDAC). Available at: http://beta.sedac.ciesin.columbia.edu/data/set/gpw-v4-population-density/. Accessed 2 February 2016.
5. Robinson TP, Wint GRW, Conchedda G, Van Boeckel TP, Ercoli V, Palamara E, et al. Mapping the global distribution of livestock. PLoS One **2015**; 9(5):e96084.
6. Gilbert M, Conchedda G, Van Boeckel TP, Cinardi G, Linard C, Nicolas G, et al. Income disparities and the global distribution of intensively farmed chickens and pigs. PLoS One **2015**; 10(7):e0133381.
7. Paul M, Wongnarkpet S, Gasqui P, Poolkhet C, Thongratsakul S, Ducrot C, et al. Risk factors for highly pathogenic avian influenza (HPAI) H5N1 infection in backyards chicken farms, Thailand. Acta Trop **2011**; 118(3):209-216.
8. Paul M, Tavornpanich S, Abrial D, Gasqui P, Charras-Garrido M, Thanapongtharm, W, et al. Anthropogenic factors and the risk of highly pathogenic avian influenza H5N1: prospects from a spatial-based model. Vet Res **2010**; 41(3):28.
9. Biswas PK, Christensen JP, Ahmed SSU, Das A, Rahman MH, Barua H, et al. Risk infection for infection with highly pathogenic avian influenza virus (H5N1) in backyard chickens, Bangladesh. Emerg Infect Dis **2009**; 15(12):1931-1936.
10. Ward MP, Maftei DN, Apostu CL, Suru AR. Association between outbreaks of highly pathogenic avian influenza subtype H5N1 and migratory waterfowl (family: *Anatidae*) populations. Zoonoses Public Health **2008**; 56:1-9.
11. Kummu M, de Moel H, Ward PJ, Varis O. How close do we live to water? A global analysis of population distance to freshwater bodies. PLoS One **2011**; 6(6):e20578
12. Gilbert M, Pfeiffer, DU. Risk factor modelling of the spatio-temporal patterns of highly pathogenic avian influenza (HPAIV) H5N1: a review. Spat Spatiotemporal Epidemiol **2012**; 3:173-183.
13. Hijmans, RJ, Cameron SE, Parra JL, Jones PG, Jarvis A. Very high resolution interpolated climate surfaces for global land areas. [Int J Climatol **2005**; 25:1965-1978](http://onlinelibrary.wiley.com/doi/10.1002/joc.1276/pdf).
14. Nelson A. Estimated travel time to the nearest city of 50,000 or more people in year 2000. Global Environmental Monitoring Unit. Ispra: Joint Research Centre of the European Commission. 2008. Available at: http://forobs.jrc.ec.europa.eu/products/gam/. Accessed 11 April 2015.
15. Gilbert M, Newman SH, Takekawa JY, Loth L, Biradar C, Prosser DJ, et al. Flying over an infected landscape: distribution of highly pathogenic avian influenza H5N1 risk in South Asia and satellite tracking of wild waterfowl. Ecohealth **2011**; 7(4):448-458.
16. Hosseini P, Sokolow SH, Vandegrift KJ, Kilpatrick AM, Daszak P. Predictive power of air travel and socio-economic data for early pandemic spread. PLoS One **2010**; 5(9):e12763.
17. De Groeve T, Vemaccini L, Pljan Pljanšek K. Index for risk management – INFORM – concept and methodology, version 2015. European Commission -Joint Research Centre. Luxembourg: Publications Office of the European Union, **2015**; 96 pp.
18. Organisation for Economic Co-operation and Development, European Commission - Joint Research Centre. Handbook on constructing composite indicators: methodology and user guide. Paris: OECD Publishing, **2008**.
19. United States Agency International Development. Design and use of composite indices in assessments of climate change vulnerability and resilience: African and Latin American resilience to climate change. USAID, **2014**.
20. Tan J, Kuchibhatla D, Sirota FL, Sherman WA, Gattermayer T, Kwoh CY, et al. Tachyon search speeds up retrieval of similar sequences by several orders of magnitude. Bioinforma Oxf Engl **2012**; 28(12):1645–6.
21. Cumulative number of confirmed human cases for avian influenza A(H5N1) reported to WHO, 2003-2015. Available at: http://www.who.int/influenza/human_animal_interface/EN_GIP_20151113cumulativeNumberH5N1cases.pdf?ua=1. Accessed 18 November 2015.
22. Richard M, de Graaf M, Herfst S. Avian influenza A viruses: from zoonosis to pandemic. Future Virol **2014**; 9(5):513–24.
23. Freidl GS, Meijer A, de Bruin E, de Nardi M, Munoz O, Capua I, et al., FLURISK Consortium. Influenza at the animal-human interface: a review of the literature for virological evidence of human infection with swine or avian influenza viruses other than A(H5N1). Euro Surveill Bull Eur Sur Mal Transm Eur Commun Dis Bull **2014**; 19(18).
24. Reperant LA, Kuiken T, Osterhaus ADME. Adaptive pathways of zoonotic influenza viruses: from exposure to establishment in humans. Vaccine **2012**; 30(30):4419–34.
25. Bastien N, Antonishyn NA, Brandt K, Wong CE, Chokani K, Vegh N, et al. Human infection with a triple-reassortant swine influenza A(H1N1) virus containing the hemagglutinin and neuraminidase genes of seasonal influenza virus. J Infect Dis **2010**; 201(8):1178–82.
