# Supplementary figures and images for: The Geographic Variation of Surveillance and Zoonotic Spillover Potential of Influenza Viruses in Domestic Poultry and Swine
Source: Open Forum Infect Dis. 2018 Nov 27;5(12):ofy318. doi: 10.1093/ofid/ofy318 (PMC6309522; doi:10.1093/ofid/ofy318)

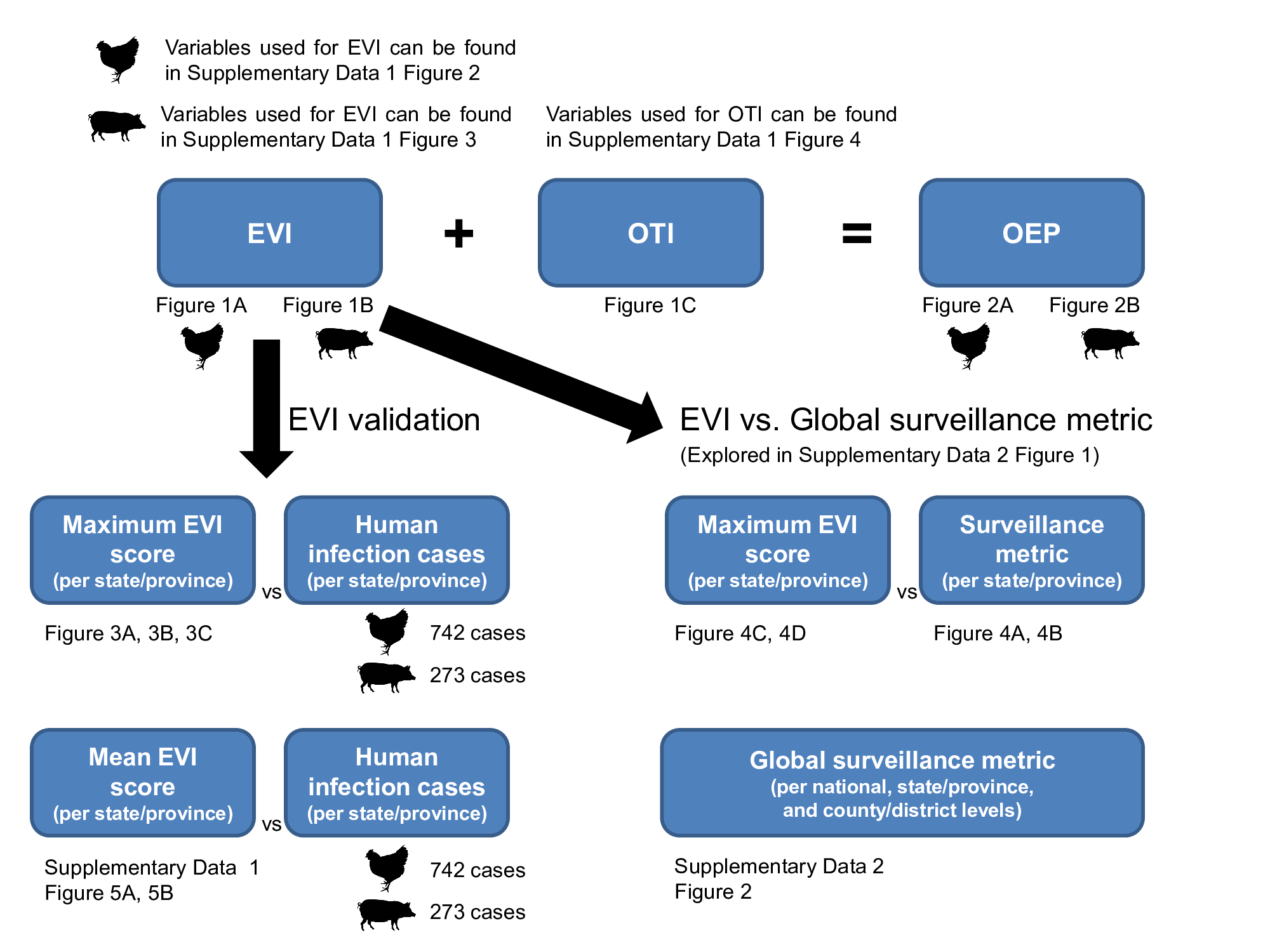

Supplement: ofy318_suppl_supplementary_data1_figure_1 [file ofy318_suppl_supplementary_data1_figure_1.png]

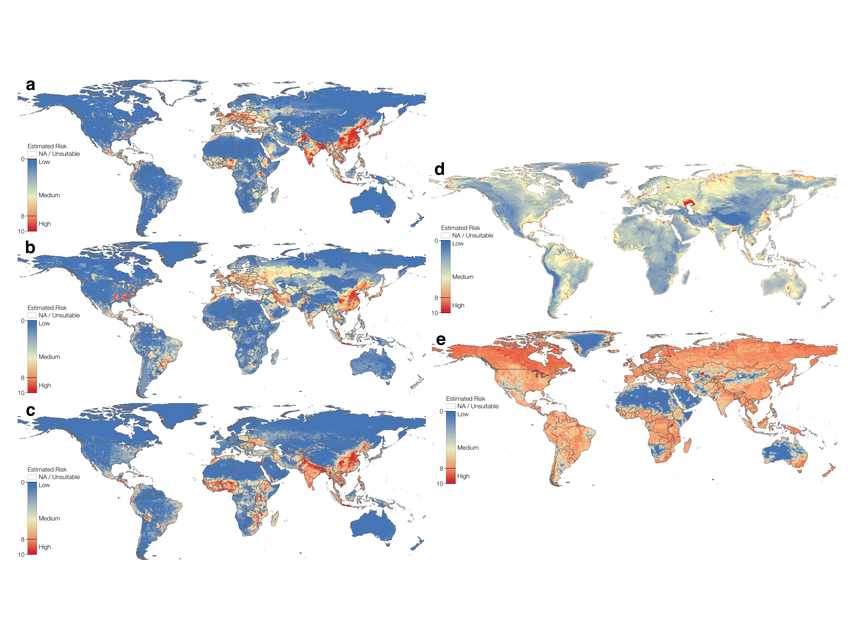

Supplement: ofy318_suppl_supplementary_data1_figure_2 [file ofy318_suppl_supplementary_data1_figure_2.png]

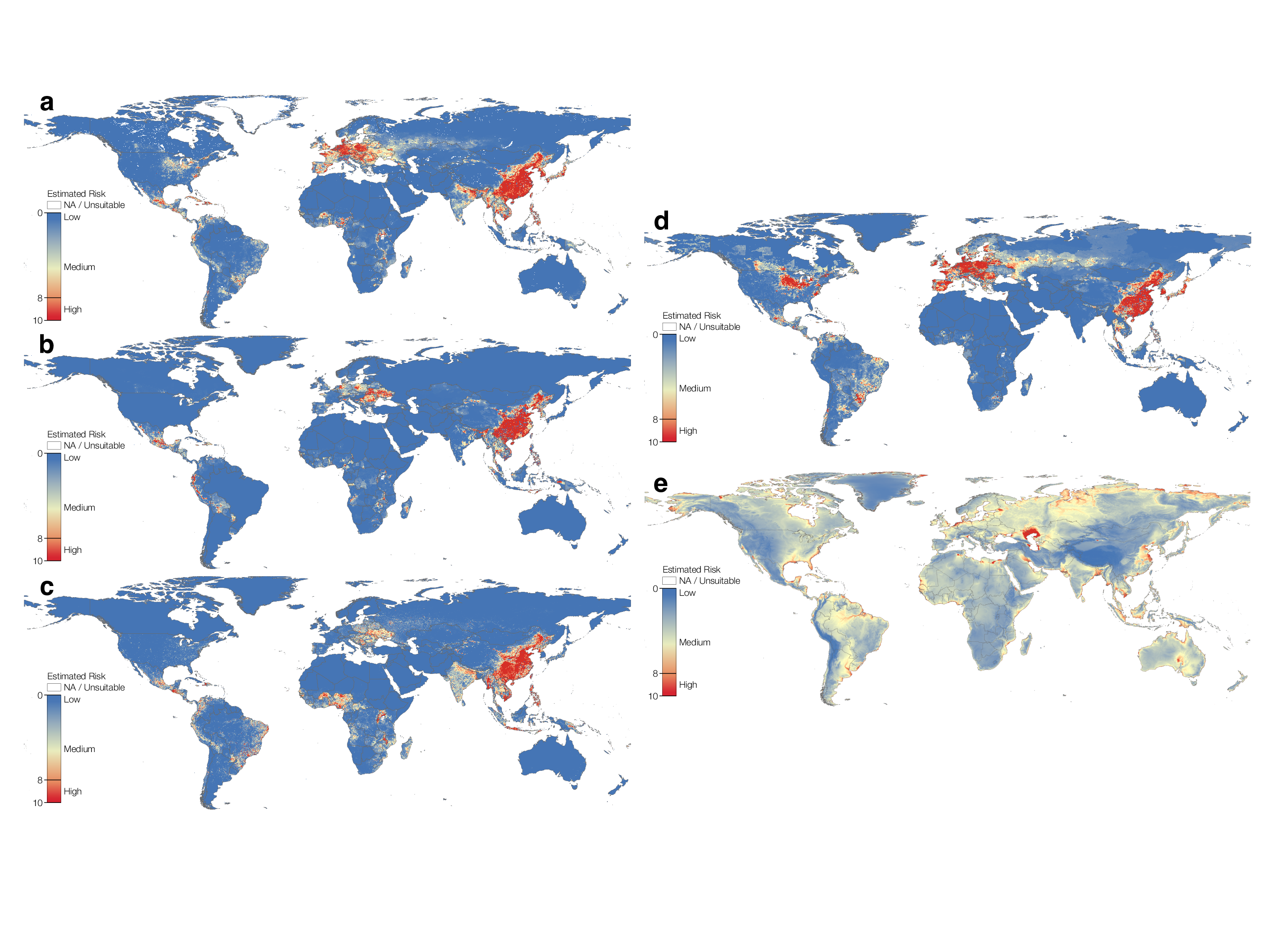

Supplement: ofy318_suppl_supplementary_data1_figure_3 [file ofy318_suppl_supplementary_data1_figure_3.png]

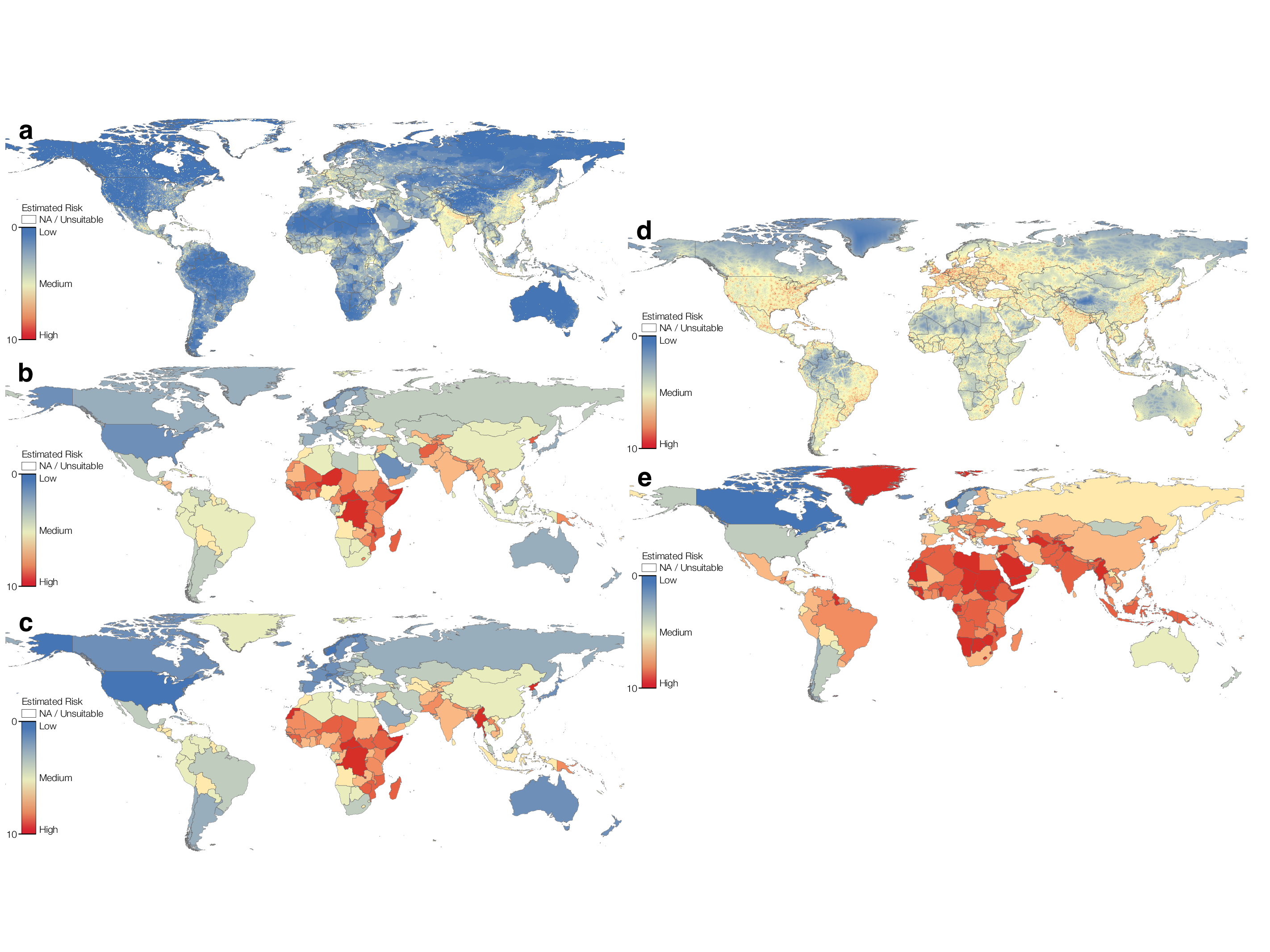

Supplement: ofy318_suppl_supplementary_data1_figure_4 [file ofy318_suppl_supplementary_data1_figure_4.png]

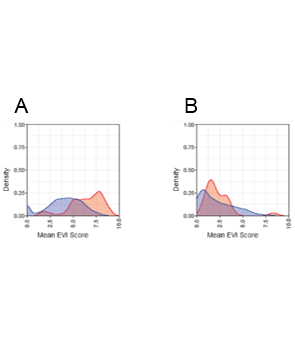

Supplement: ofy318_suppl_supplementary_data1_figure_5 [file ofy318_suppl_supplementary_data1_figure_5.png]

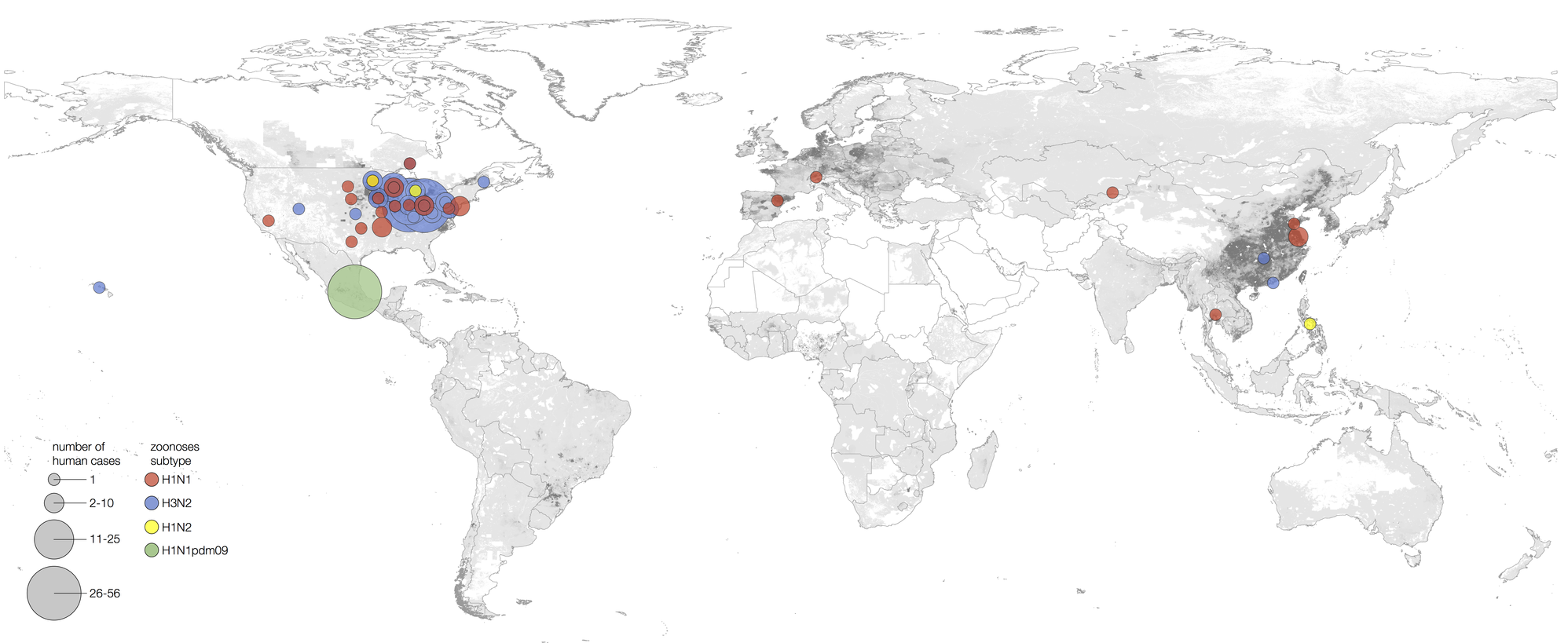

Supplement: ofy318_suppl_supplementary_data1_figure_6 [file ofy318_suppl_supplementary_data1_figure_6.png]

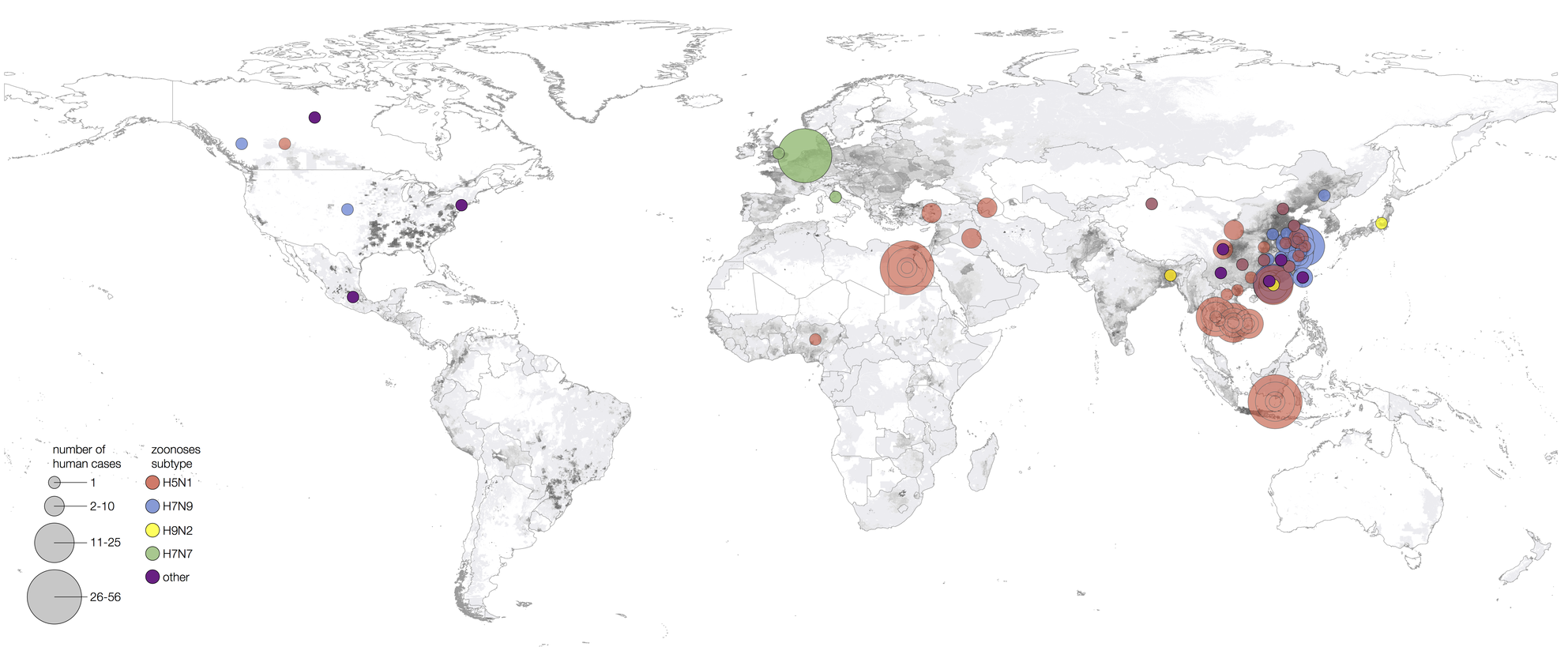

Supplement: ofy318_suppl_supplementary_data1_figure_7 [file ofy318_suppl_supplementary_data1_figure_7.png]

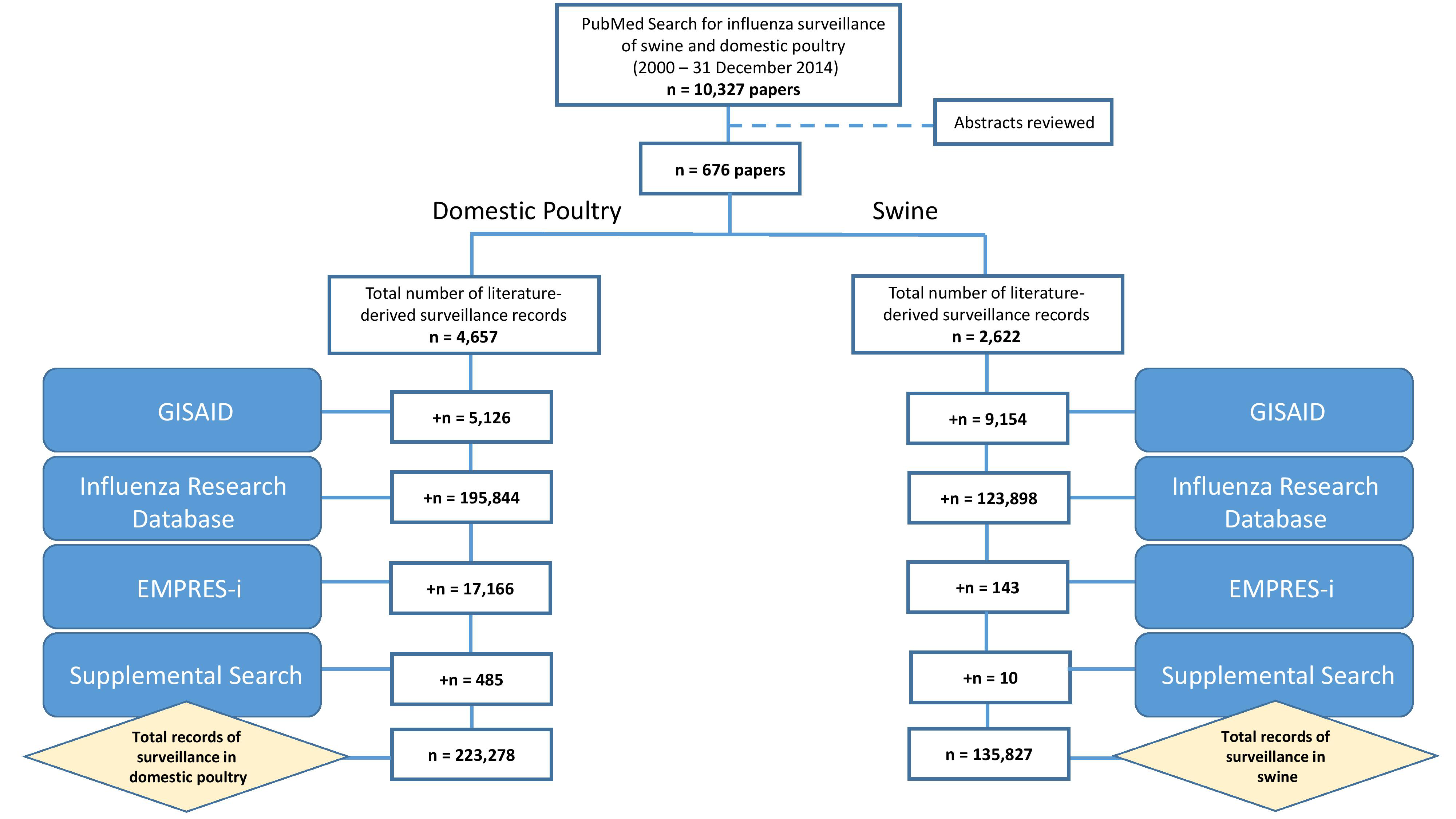

Supplement: ofy318_suppl_supplementary_data2_figure_1 [file ofy318_suppl_supplementary_data2_figure_1.png]

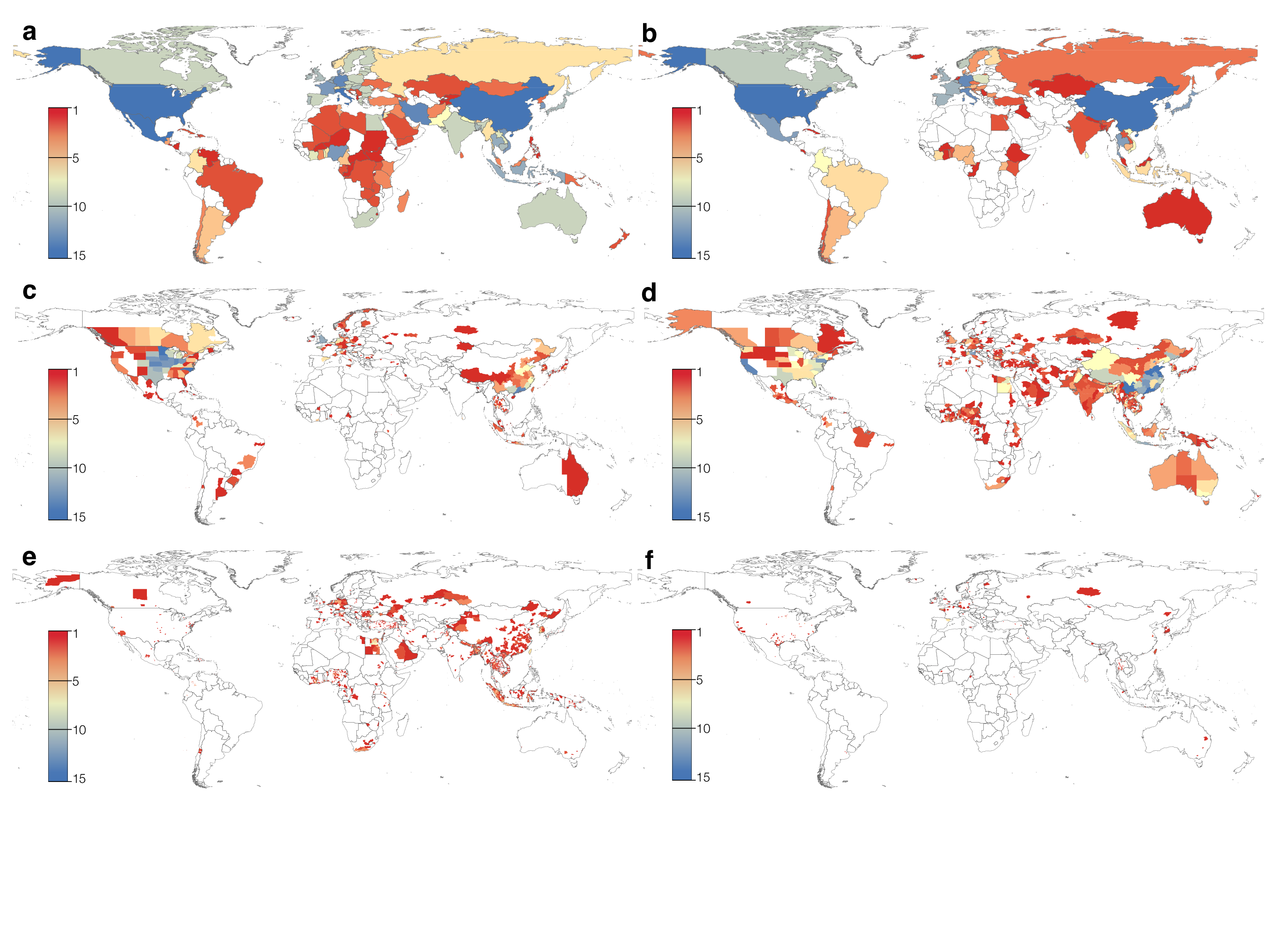

Supplement: ofy318_suppl_supplementary_data2_figure_2 [file ofy318_suppl_supplementary_data2_figure_2.png]
