# Supplementary material for: The Geographic Variation of Surveillance and Zoonotic Spillover Potential of Influenza Viruses in Domestic Poultry and Swine
Source: Open Forum Infect Dis. 2018 Nov 27;5(12):ofy318. doi: 10.1093/ofid/ofy318 (PMC6309522; doi:10.1093/ofid/ofy318)
Supplement: ofy318_suppl_supplementary_data2 [file ofy318_suppl_supplementary_data2.docx]

**SUPPLEMENTARY DATA 2**

**The geographic variation of surveillance and zoonotic spillover potential of influenza viruses in domestic poultry and swine**

Kathryn A. Berger, David M. Pigott, Francesca Tomlinson, David Godding, Sebastian Maurer-Stroh, Biruhalem Taye, Fernanda L. Sirota, Alvin Han, Raphael T.C. Lee, Vithiagaran Gunalan, Frank Eisenhaber, Simon I. Hay, Colin A. Russell

**SUPPLEMENTARY METHODS**

**Global animal influenza surveillance**

To develop the surveillance metric presented in the manuscript, we assembled a global database of viral genetic sequences, records of influenza-positive animals, and data from formal surveillance initiatives recorded between January 2000 and December 2014. A total of 5 sources were used in the development of this global surveillance database (see Supplementary Data 2 Figure 1). Here we provide additional detail on the search terms and criteria used in its development.

1. **Literature**

Two separate systematic literature reviews were performed to identify global surveillance programs for both domestic poultry and swine between 2000 and 2014. The PRISMA [1] methodology was utilized, with both literature reviews restricted to the English language and last updated in December 2014. A search through the PubMed database (http://ncbi.nlm.nih.gov/entrez/query.fcgi) for the key terms ‘influenza’ and references to swine (i.e., ‘boar’, ‘pig’, ‘pigs’, ‘scrofa’, or ‘swine’) or domestic poultry (i.e., ‘chicken’, ‘duck’, ‘poultry’, or ‘turkey’) were used to identify relevant articles. An additional search criterion was added to include geographic location, including both continent and any variation on country name notation (e.g., USA, United States of America). Each search term was designed to return the largest number of relevant results and performed on a continental basis due to the substantial number of titles. Example search term:

((avian influenza) AND ((((turkey) OR chicken) OR poultry) OR duck)) AND ((((((((((((((((((((((((((((((((((((((((((((((((((((((europe) OR russia) OR ukraine) OR france) OR spain) OR sweden) OR norway) OR germany) OR finland) OR poland) OR italy) OR united kingdom) OR britain) OR england) OR scotland) OR wales) OR romania) OR belarus) OR kazakhstan) OR greece) OR bulgaria) OR iceland) OR hungary) OR portugal) OR serbia) OR austria) OR czech republic) OR ireland) OR lithuania) OR latvia) OR croatia) OR bosnia) OR slovakia) OR estonia) OR denmark) OR netherlands) OR switzerland) OR moldova) OR belgium) OR albania) OR macedonia) OR turkey) OR slovenia) OR montenegro) OR cyprus) OR azerbaijan) OR luxembourg) OR georgia) OR andorra) OR malta) OR liechtenstein) OR san marino) OR monaco) OR vatican city).

All titles found within search results were then screened based on six exclusion criteria:

1. *Not on influenza*
2. *Study prior to 2000*
3. *Incorrect continent*
4. *Not relating to domesticated swine or wild boar*
5. *Describes experimental study rather than surveillance*
6. *Not peer reviewed (i.e., news story reporting on published work)*

If results could not be excluded based on title alone, abstracts were then read and processed through the same applied criteria. All peer-reviewed articles that passed through this final stage of systematic review process were designated for further reading and assessed for relevance. Articles that did not contain information on animal influenza surveillance activity were excluded from analysis.

1. **Animal influenza Sequences**

Archived virus isolate sequence data derived from animal hosts were downloaded from GISAID, a publicly accessible dataset with registration, online (http://platform.gisaid.org/epi3/frontend#45f145). All records for Influenza A viruses were selected for all animal host species and for all locations. Records containing as little as one gene segment were retained for analysis and were filtered from the dates 01 January 2000 to 31 December 2014. The following selection criteria were further applied to filter out records for domestic poultry and swine:

1. *For domestic poultry*: "Host" = 'chicken' OR "Host" = 'Chicken' OR "Host" = "Ch" OR "Host" = 'domestic duck' OR "Host" = 'domestic goose' OR "Host" = 'fowl' OR "Host" = 'Fowl' OR "Host" = 'Gallus gallus' OR "Host" = 'goose' OR "Host" = 'Goose.' Please note that the following search term 'Host' = 'Turkey' OR 'Host' = 'turkey' was excluded from the above query because there was no way to ascertain whether these records were referring to domestic or wild turkeys. If these search terms were included into the analysis, it would have resulted in an additional 1,026 records. Likewise, if the terms "Host" = 'Avian' OR 'Host' = 'avian' were included, we would have included an additional 1,970 records.
2. *For swine*: "Host" = 'swine' OR "Host" = 'Swine. '
3. **Animal influenza surveillance programs**

Global influenza surveillance data collected by NIAID-funded CEIRS initiatives, for both avian non-human mammal hosts, were downloaded from Influenza Research Database (IRD), a publicly accessible dataset available online (http://www.fludb.org). Records for domestic poultry were derived by selecting all tested samples under surveillance data type ‘avian’, including all host species and displaying all surveillance samples. Using this selection criterion we then extracted those records where both host name and bird behaviour suggested inclusion into our database. Due to data entry discrepancies within the IRD database itself, special care was taken to examine the columns for ‘Host Common Name’, ‘Scientific Name’ and ‘Bird Behaviour’ to be all inclusive; as the selection of only ‘Host Common Name’ might have precluded birds described as domestic species (e.g., Host Common Name: ‘Duck’; Scientific Name: ‘Anser anser’; Bird Behaviour: ‘Domestic’). Likewise, records for swine were derived by selecting all tested samples under surveillance data type: ‘Non-human mammal’ including all host species and displaying all surveillance samples. From this selection, all records with host species attributes for ‘swine’ and ‘wild boar’ were selected for inclusion into our surveillance database for swine. The following selection criteria were applied to filter out records for domestic poultry and swine:

1. *For domestic poultry*: Host_Common_Name = 'Chicken' OR Host_Common_Name = 'Chicken Or Pheasant' OR Host_Common_Name = 'Chicken Or Rock Pigeon' OR Host_Common_Name = 'Domestic Chicken' OR Host_Common_Name = 'DOMESTIC DUCK' OR Host_Common_Name = 'Domestic Goose' OR Host_Common_Name = 'DOMESTIC GOOSE' OR Host_Common_Name = 'Domestic Pigeon' OR Host_Common_Name = 'Greylag Goose (Domestic)' OR Host_Common_Name = 'Mallard (Domestic).' Furthermore due to differences in data entry the attribute 'Bird Behaviour' was queried for all records labelled 'Domestic', of which the following species names were then selected: 'meleagaris gallopavo' OR 'meleagris sp.'; (Geese) 'Anser anser' OR 'Anser anser (var domesticus)' OR 'anser anser domesticus' OR 'anser cygnoides' OR 'anser sp.'; (Ducks) 'Anas platyrhynchos' OR 'Anas platyrhynchos [var domesticus]' OR 'Anas platyrhynchos domesticus' OR 'Anas sp' OR 'Anas sp.' OR 'Anas spp' OR 'Anas spp.' OR 'cairina moschata' OR 'Cairina moschata x Anas platyrhynchos'; (Chicken) 'gallus domesticus' OR 'gallus gallus domesticus' OR 'Gallus sp.' (Chukar) 'Alectoris chukar' OR 'Alectoris chukar/Columbia livia' OR 'Alectoris chukar/Phasiarus colchicus'; Quail [Japanese] 'Corturnix japonica' OR 'Corturnix sp.'
2. *For swine*: The search terms 'swine' and 'wild boar' were selected from 'Host_Common_Name.'
3. **Influenza-positive animals**

Epidemiological reports of animal influenza events were derived from the FAO’s EMPRES-I database, where they are available for public download (http://empres-i.fao.org/eipws3g/). All records were downloaded from the ‘Disease Events’ tab listed and listed under ‘Influenza – Avian’ and ‘Influenza – Swine’, respectively. The attribute ‘Species Description’ was used to determine inclusion of records into its classification under domestic poultry or swine surveillance. The following selection criteria were later applied to filter out records for domestic poultry and swine:

1. *For domestic poultry*: Select by attribute terms for the file (EMPRES_domestic_captive). 'Species_De' LIKE 'domestic%' OR 'Species_De' LIKE 'captive%' AND 'Species_De' LIKE 'chi%' OR 'duck%' or 'bird%'. From this selected list of results, steps were taken to remove those that included non-domesticated poultry (i.e., 'captive tiger') using the SQL terms: Select *FROM EMPRES_domestic_captive where: 'Species_De' NOT IN ('domestic, cats', 'domestic, dogs', 'domestic donkey', 'domestic ferret', 'domestic, horse', 'domestic mink', 'domestic, swine').
2. *For swine*: Search terms selected included 'domestic swine' or 'swine' were included within the animals listed under header 'Species_De' during each outbreak. Importantly, EMPRES records draw from the OIE notification system, where reporting of outbreaks in swine is not mandated.
3. **Supplemental web search**

To identify surveillance efforts that were not captured by the search described above, we performed additional internet searches (Google, confined to English language results) with keywords ‘domestic poultry’, ‘chicken’, ‘swine’, ‘pig’, ‘influenza’, and ‘surveillance’ followed by ‘country name’. These searches were performed for all countries worldwide. This search identified five additional surveillance initiatives not identified through other data sources (Supplementary Data 2 Table 1).

1. **Geo-positioning and curation of surveillance data**

All surveillance data were downloaded and last updated in December 2014. Geo-positioning of all records ranged from point (precise location with latitude/longitude coordinates), to areas (polygons) corresponding to GAUL administrative boundary areas. Geographic information was manually extracted from each peer-reviewed reference identified from the systematic PubMed literature search. In many cases, either due to multiple locations sharing the same name, or due to differences in spelling (e.g., Naples versus Napoli), further contextual information was used to ensure classification to the correct location. As our global database was compiled from a variety of disparate data streams (Supplementary Data 2 Figure 1), it was essential to standardize all entries so that identical locations were geo-positioned using the same unique GAUL identifier. Due to the nature of dealing with incongruent databases and a multitude of temporal forms, all temporal data were standardized into a single format to read MM-DD-YYYY. As some data described transmission or surveillance activities across multiple years, records were reported for each unique location, for each calendar year. This resulted in the disaggregation of records in the same location spanning multiple years into individual records for each unique location for each respective year.

**RESULTS**

**Surveillance Metric Mapping**

A final global database consisting of 359,105 surveillance records, from 128 countries, between 2000 and 2014, was constructed following the methodology described above. Over half of all surveillance records where for domestic poultry, with the spatial resolution of each record decreasing from national to sub-national administrative boundaries (see Supplementary Data 2 Table 2). The key exception being the extremely high number of surveillance records recorded at the point (lat/long coordinates) level for domestic poultry. Many of these records (195,844 out of 223,278) were reported within the IRD database, where formal surveillance programs recorded coordinates for each sample’s location. It should be noted, however, that while IRD records make up the 87.71% of domestic poultry (195,844 out of 223,278) records and the 91.22% (123,898 out of 135,817) of swine surveillance records, IRD surveillance initiatives are established in only 32 countries worldwide. Here we present the spatial and temporal variation in global animal influenza surveillance efforts, as a result of our surveillance metric (Supplementary Data 2 Figure 2).

**Supplementary Data 2 Table 1**. Supplemental surveillance search results at the national scale for both domestic poultry and swine.

| Country | Domestic poultry | Swine |
| --- | --- | --- |
| Australia | Australian National Avian Influenza Surveillance Dossier  *Source* : OCVO (2010) National Avian Influenza Surveillance Dossier. Office of the Chief Veterinary Officer, Australian Government Department of Agriculture, Fisheries and Forestry, Canberra. |  |
| European Commission | European Commission -Annual Reports  *Source*: (<http://ec.europa.eu/food/animals/animal-diseases/control-measures/avian-influenza_en>) |  |
| Norway | Norwegian Veterinary Institute - Annual Reports  *Source*: (<http://wwweng.vetinst.no/eng/index.html>) | Norwegian Veterinary Institute - Annual Reports  *Source*:  (<http://wwweng.vetinst.no/eng/index.html>) |
| United States of America | United States Department of Agriculture – Summary and Quarterly Reports  *Source*:  (<https://www.aphis.usda.gov/aphis/ourfocus/animalhealth/animal-disease-information/avian-influenza-disease/sa_avian_influenza/ct_avian_influenza_surveillance_meat_type_chickens>) |  |

**Supplementary Data 2 Table 2**. A description of a global database of animal influenza surveillance activities by species and spatial detail.

| Species | | National  (GAUL_0) | State/Province  (GAUL_1) | District/County  (GAUL_2) | | Point  (Lat/Long) | | Total  Records |  |
| --- | --- | --- | --- | --- | --- | --- | --- | --- | --- |
| Domestic Poultry | | 4,397 | | 5,326 | 1,340 | 212,215 | | 223,278 | |
| Swine | | 33,704 | | 87,616 | 4,741 | 9,766 | | 135,827 | |
| Total | | 38,101 | | 92,942 | 6,081 | 221,981 | | 359,105 | |

**Supplementary Data 2 Figure Legends**

**Supplementary Data 2 Figure 1**. Conceptual diagram of the assembly of our global animal influenza surveillance database.

**Supplementary Data 2 Figure 2**. Global records of surveillance across all geospatial scales. Domestic poultry at surveillance at the national (A), state/province (B), and county/district levels (C). Swine surveillance at the national (D), state/province (E), and county/district levels (F). Colder colors demonstrating greater years of surveillance (blue = 15 years of surveillance); warmer colors indicating minimal surveillance (red = 1 year of surveillance).

**REFERENCES**

1. Moher D, Liberati A, Tetzlaff J, Altman DG, The PRISMA Group. Preferred reporting items for systematic reviews and meta-analyses: the PRISMA statement. PLoS Med **2009**; 6(7):e1000097.
